# *Bartonella henselae* in Skin Biopsy Specimens of Patients with Cat-Scratch Disease

**DOI:** 10.3201/eid1612.100647

**Published:** 2010-12

**Authors:** Emmanouil Angelakis, Sophie Edouard, Bernard La Scola, Didier Raoult

**Affiliations:** Author affiliation: Université de la Méditerranée, Marseille, France

**Keywords:** Bartonella henselae, skin biopsy, cat-scratch disease, zoonoses, bacteria, France, dispatch

## Abstract

During the past 2 years, we identified live *Bartonella henselae* in the primary inoculation sites of 3 patients after a cat scratch. Although our data are preliminary, we report that a cutaneous swab of the skin lesion from a patient in the early stage of cat-scratch disease can be useful for diagnosis of the infection.

*Bartonella henselae* is the main causative agent of cat-scratch disease (CSD). Little is known about the organism’s pathogenesis in long-lasting lymphadenopathy, but an immunopathogenesis is assumed (*1*). *B. henselae* is infrequently grown from the lymph nodes of humans, and only in a few cases was *B. henselae* isolated from patients with CSD (*2**,**3*). In experiments with mice, *B. henselae* was eliminated within a few days to 1 week after systemic (intraperitoneal or intravenous) infection (*4*). Moreover, on the basis of molecular methods, we recently identified that the scalp eschars from 2 patients who were bitten by a tick contained *B. henselae* (*5*). In this study, our objective was to determine if *B. henselae* was present in the papule, which is developed in the scratch line. We report isolation of *B. henselae* from a swab specimen and the skin biopsy specimens sampled from the skin papule of 3 patients with CSD.

## The Study

From January 2007 through February 2010, we tested 92 skin biopsy specimens from patients suspected of having CSD. DNA was extracted by using a QIAamp Tissue Kit (QIAGEN, Valencia, CA, USA) and was used as a template in a previously described real-time reverse transcription–PCR (RT-PCR) specific for a portion of the *Bartonella* 16S–23S intergenic spacer region and the *PAP31* gene for detection of *B. henselae* (*6*). *B. henselae* was identified in 4 skin biopsy specimens (4.3%). For each patient, we received a skin biopsy specimen from the skin papule, a lymph node biopsy specimen, and paired serum samples. For 1 patient, we also received a swab from a skin papule. Immunoglobulin G and M titers were determined by using an immunofluorescent antibody assay (*7*).

Skin biopsy specimens and the swab were cultured in human embryonic lung fibroblasts by using the centrifugation shell-vial technique (3.7 mL; Sterilin Ltd., Felthan, UK); 12-mm round coverslips seeded with 1 mL of medium containing 50,000 cells and incubated in a 5% CO_2_ incubator at 37°C for 3 days were used to obtain a confluent monolayer (*8*). Cultures were surveyed for 4 weeks and detection of bacteria growth was assessed every 7 days on coverslips directly inside the shell vial by using Gimenez and immunofluorescence staining. We obtained a positive culture from 3 patients, and detailed histories are described below (Table).

**Table:** Assessment and testing results for 3 patients with cat scratch disease who had skin biopsy specimens positive for *Bartonella henselae*, France, 2010*

Patient no.	Fever	Lymphadenopathy	Cat contact	Serologic results for *Bartonella* spp.	RT-PCR results for *B. henselae*				Culture results (incubation period)	
Skin biopsy	Lymph node	Swab		Skin biopsy	Swab
1	Yes	Right auxiliary and epitrochlear lymphadenitis	Yes	Positive	Yes	Yes	ND		Positive (2 wk)	ND
2	No	Right inguinal and epitrochlear lymphadenitis	Yes	Positive	Yes	Yes	Yes		Positive (3 wk)	Positive (3 wk)
3	No	Left auxiliary lymphadenitis	Yes	Positive	Yes	Yes	ND		Positive (2 wk)	ND

*RT-PCR, reverse transcription–PCR; ND, not done.

Patient 1 was a 38-year-old man who had fever (40°C) and asthenia. The patient was a cat owner who had been scratched 8 days before onset of symptoms. Clinical signs were right axillary lymphadenitis and an inflammatory reddish skin lesion on the right hand with epitrochlear adenopathy, which appeared 2 days before he sought treatment. Abdominal ultrasound showed small hepatic abscesses. After the skin biopsy sample was obtained, doxycycline (200 mg/d) was given for 1 week. The patient fully recovered.

Patient 2 was a 17-year-old man with an inflamed red skin lesion on the right foot and epitrochlear adenopathy. The patient reported that he was scratched ≈1 week earlier by his cat and that the skin lesion appeared the day before he sought treatment. Right inguinal lymphadenitis was also identified during examination.

Patient 3 was a 20-year-old man had an inflammatory skin lesion on the left hand. He had a cat scratch 9 days before; the skin papule appeared 1 day before he sought treatment. Left axilliary lymphadenitis was identified during the examination and abdominal ultrasound showed hepatomegaly.

Skin biopsy specimens and lymph nodes from all patients were positive by real-time RT-PCR; patient 2 also had a positive swab specimen. Moreover, all patients had serum samples positive for *B. henselae* by immunofluorescent antibody assay. We detected gram-negative bacilli (Figure), which were identified as *B. henselae* by real-time RT-PCR (*6*), in the cultures of the skin biopsies and swab specimen. Patients 2 and 3 recovered without treatment.

**Figure:**
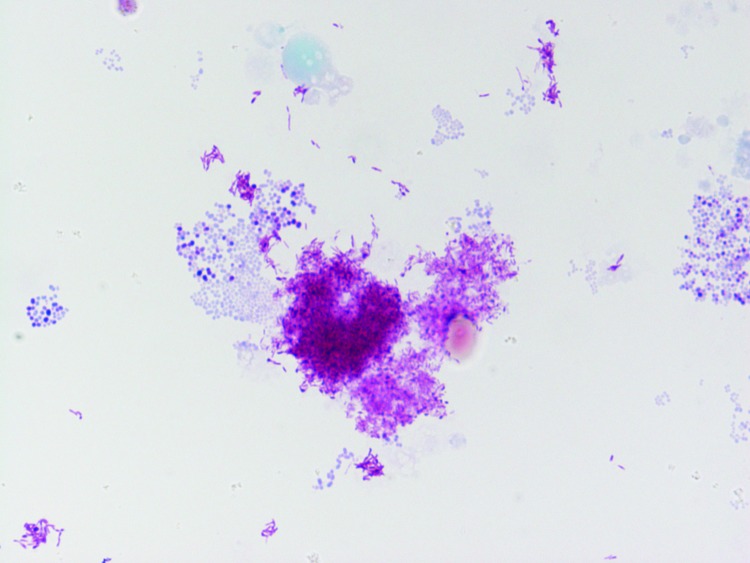
Gimenez stain of *Bartonella henselae* obtained by the culture in human embryonic lung of the skin biopsy of a patient with cat scratch disease, France, 2010. Original magnification ×100.

## Conclusions

We isolated *B. henselae* from skin biopsy specimens of 3 patients with CSD. Patients with CSD usually have gradual regional lymph node enlargement, accompanied by a papule, which develops in the scratch line after 3–10 days; the papule may persist for only a few days or as long as 2–3 weeks (*9*). Histopathologic appearance of the skin lesion is similar to lymph node changes, consisting of a diffuse inflammatory cell infiltrate associating numerous neutrophils and histiocytes mixed with scattered eosinophils and plasma cells (*9*). Other more unusual skin manifestations include morbilliform eruptions, urticaria, erythema nodosum, erythema multiforme, and erythema marginatum (*9*). *B. henselae* in the skin papule was first proposed by Wear et al., who reported that the primary inoculation site and the lymph nodes of patients with CSD contained the same small Gram-negative bacilli (*10*). Using immunohistochemical stain, Lin et al. found *B. henselae* in the cytoplasm of histiocytes within the granulomatous lesions in 9 lymph nodes and 1 skin biopsy specimen from patients with CSD (*11*). Avidor et al. identified *B. henselae* in inflammatory papules and pustules of 2 patients with CSD (*12*). Our group recently identified *B. henselae* in patients with scalp eschars and neck lymphadenopathy after tick bites (*5*). Moreover, Fournier et al. detected *B. henselae* in 2 skin biopsy specimens of a primary papule from patients in Australia clinically suspected of having CSD (*2*).

Swabs of lesions for the diagnosis and culture of *B. henselae* are not widely used. Fournier et al. found that swabs from 6 primary skin papules from patients clinically suspected of having CSD were positive for *B. henselae*; a positive culture was also obtained from 1 cutaneous swab (*2*). For rickettsial diseases, in 2006, the diagnosis of 1 case of scrub typhus was based on PCR results of the patient’s eschar (*13*). Wang et al. identified 3 cases of Queensland tick typhus caused by *Rickettsia australis* and 1 case of African tick bite fever caused by *R. africae* by the use of PCR in dry and sterile saline moistened swabs collected from the eschar margin (*14*). *B. henselae* is often isolated from cutaneous tumors in AIDS and immunocompromised patients with bacillary angiomatosis (*15*); however, all our patients were immunocompetent.

In conclusion, we found live *B. henselae* in the primary inoculation site after a cat scratch. An incubation period of 2–3 weeks was necessary to obtain *B. henselae* isolates from the skin lesion, therefore, cultures are not proposed for point-of-care diagnosis. To reduce the delay in diagnosis, real-time RT-PCR enables rapid detection and identification of CSD in skin biopsy specimens and swabs. Probably crucial for the isolation of *B. henselae* was the fact that the skin biopsy specimens and the swab were sampled early after appearance of the skin papule and that patients did not receive treatment. Two of 3 patients recovered without antimicrobial drug treatment, which leads us to believe that treatment with antimicrobial drugs is not necessary for immunocompetent patients. A cutaneous swab of the skin lesion in the early stage of CSD infection may replace the more painful skin or lymph node biopsies.
